# Erratum

**Published:** 2023-01

In manuscript “Invoking the in uence of emotion in central auditory processing to improve the treatment of speech impairments” Saudi Med J 2021; Vol. 42 (12): 1325-1332 doi: 10.15537/smj.2021.42.12.20200724. e disclosure section should appeared as: is study was funded by Jordan University of Science and Technology, Irbid, Jordan. Grant No: 20190016.

